# Whole-body MRI, healthcare ethics and access equity: Where are we? Where are we going?

**DOI:** 10.1093/radadv/umag027

**Published:** 2026-06-12

**Authors:** Ariadne Maria Krzyuy, Leonardo C Welling, Mário Augusto Cray da Costa

**Affiliations:** Postgraduate Program in Health Sciences, State University of Ponta Grossa, Ponta Grossa, Paraná, CEP 84030-900, Brazil; Postgraduate Program in Health Sciences, State University of Ponta Grossa, Ponta Grossa, Paraná, CEP 84030-900, Brazil; Department of Neurological Surgery, State University of Ponta Grossa, Ponta Grossa, Paraná, Brazil; Postgraduate Program in Health Sciences, State University of Ponta Grossa, Ponta Grossa, Paraná, CEP 84030-900, Brazil; Department of Medicine, State University of Ponta Grossa, Ponta Grossa, Paraná, Brazil

**Keywords:** whole-body MRI, ethical considerations, screening program

We read with great interest the article “How to implement a radiologist-led whole-body MRI screening program.”^1^ The manuscript supplies a structured proposal for implementing whole-body magnetic resonance imaging (WB-MRI) as a screening strategy. However, from the perspectives of the health sciences and the philosophy of medicine, several important points call for further consideration.

Screening asymptomatic individuals with WB-MRI has some limitations. While cancer identification rates are 1%–2%, incidental findings are observed in up to 97% of cases. This disproportion elicits significant questions about whether relevant diseases are being recognized or creating diagnostic uncertainty, especially regarding their natural history.^2^

It should be observed that overdiagnosis carries ethical issues. Especially when irrelevant findings are demonstrated, the healthy individuals can become patients, subjecting them to complementary tests, unnecessary procedures, and psychological distress without proven survival benefits.^3^

The routine implementation of WB-MRI, particularly through direct-to-consumer models, can connect medical practice with market interests. While radiologist-led programs are positioned as an ethical alternative, strong evidence of their effectiveness in low-risk populations is still lacking.^4^

Equity of access is another important consideration. WB-MRI is typically privately funded and expensive, limiting access to those with more financial means. This situation may reinforce structural health inequalities and contradict the principle of justice.

Philosophically, the expansion of WB-MRI signals a shift from reactive to hyper-preventive medicine. While this development is promising, it may also pathologize normality and increase anxiety levels among healthy individuals.^5^

The work by Kierans et al. is a technical contribution, and we do not question that. But the ethical section of the manuscript, as written, remains supported by consent and communication, while more difficult questions go unasked. The expansion of WB-MRI in asymptomatic individuals requires the radiology community to respond not only to how to implement the examination but also whether and for whom it should be offered.

Overall, while the article contributes considerably to the operational discussion of WB-MRI, its wider implementation needs to be supported by stronger clinical evidence and a thorough ethical analysis.

Sincerely,

## Supplementary Material

umag027_Supplementary_Data
